# Quantitative MRCP as Part of Primary Sclerosing Cholangitis Standard of Care in the National Health Service in England: A Feasibility Assessment Among Hepatologists

**DOI:** 10.3390/healthcare13202630

**Published:** 2025-10-20

**Authors:** Elizabeth Shumbayawonda, Mamta Bajre, Daniel Eadle, Carlos Ferreira, Michele Pansini, Rajarshi Banerjee

**Affiliations:** 1 Perspectum Ltd., Gemini One, 5520 John Smith Drive, Oxford OX4 2LL, UK; 2 Health Innovation Oxford & Thames Valley, Oxford OX4 4GA, UK; 3 PSC Support, Unit 23056, Manchester M61 0BW, UK; 4 Clinica Di Radiologia EOC, Istituto Di Imaging Della Svizzera Italiana (IIMSI), Ente Ospedaliero Cantonale, Via Tesserete 46, 6900 Lugano, Switzerland; 5 John Radcliffe Hospital, Oxford University Hospitals NHS Foundation Trust, Oxford OX3 0AG, UK

**Keywords:** primary sclerosing cholangitis, magnetic resonance cholangiopancreatography, MRCP+, artificial intelligence

## Abstract

**Background:** Primary sclerosing cholangitis (PSC) is a rare chronic liver disease characterised by bile duct strictures. Magnetic resonance cholangiopancreatography (MRCP) is the principal imaging modality for diagnosis; however, its interpretation is subjective. Quantitative MRCP (MRCP+) provides quantitative assessment of the biliary anatomy and can support objective disease assessment. We evaluated the potential impact, feasibility, and perceived usefulness that MRCP+ would have on PSC patient management. **Methods**: Alongside systematic evaluation of UK and European clinical guidelines on PSC management, semi-structured interviews with 16 stakeholders were conducted. The Lean Assessment Process methodology was used to assess potential impact and feasibility of adopting MRCP+ for the PSC care pathway within the NHS. Price as a barrier to adoption was investigated to evaluate perceptions between technology cost and adoption. Perceived ease of use and perceived trust were calculated and used to evaluate perceived usefulness (PU). **Results**: For PSC management, MRCP (81%) scored higher than liver biopsy (68%) and ERCP (50%) due to its non-invasive nature. There was good internal consistency between responders on the relationship between price point and the use of MRCP+ to support diagnosis (CA:0.836) and monitoring (CA:0.904). A price point of up to GBP 500 was unlikely to be a barrier for adoption. The overall perceived usefulness for MRCP+ for patient management was 74%. **Conclusions**: There is strong interest in using MRCP+ to support PSC management. MRCP+ has the potential to address unmet needs including reducing subjectivity, measurement of the whole biliary tree and objectively measuring biliary disease progression.

## 1. Introduction

Primary sclerosing cholangitis (PSC) is a rare, immune-mediated chronic (lifelong) liver disease characterised by inflammation, fibrosis and strictures in the intrahepatic and/or extrahepatic bile ducts, leading to cholestasis and eventually liver cirrhosis [1,2]. Although a rare disease, with a reported prevalence of ~23.99 per 100,000 globally (between 3.04 and 4.80 per 100,000 in the UK), the incidence and prevalence of PSC are on the rise [3,4]. PSC can considerably impact patients’ long-term health and quality of life [5] due to an increased risk of complications [6], which include hepatobiliary cancers [7,8]. In addition to impaired quality of life, PSC has a substantial economic burden [9,10] to both the patient (high cost of illness, high patient-out-of-pocket costs, high quality and disability-adjusted life years [QALYs and DALYs], loss of work productivity) and healthcare systems (increased healthcare utilisation costs including access to specialist care) [11].

Despite advances in the understanding of PSC, liver transplant remains the only treatment that provides the possibility of complete remission [1,12]. However, liver transplant is associated with significant risks and recurrence of PSC is a common post-transplant complication [13,14]. A lack of quantitative surrogate biomarkers as acceptable measures of treatment efficacy is the biggest hindrance to developing early-stage pharmaceutical interventions for PSC [15,16]. This is crucial for patient management given the significant clinical heterogeneity [17,18] and often slow progression of PSC [19], with an average liver transplant-free survival of 14.5–21.3 years [20,21].

The British Society of Gastroenterology (BSG) and UK-PSC’s clinical guidelines for the diagnosis and management of patients with suspected PSC recommend magnetic resonance cholangiopancreatography (MRCP) as the principal imaging modality for diagnosis [1]. However, the interpretation of MRCP is subjective and thus suffers from high inter- and intra-operator variation [22,23,24]. Furthermore, MRCP is less sensitive to early, subtle changes in the bile ducts and less specific in patients with cirrhosis, making risk stratification challenging. Another limitation of MRCP is its inability to distinguish between benign and malignant biliary strictures [1], thus impeding the surveillance and early detection of hepatobiliary cancers (one of the primary disease progression concerns). The International PSC Study Group recently highlighted the urgent need to standardise diagnosis and treatment based on these limitations in diagnostic imaging techniques in PSC, including MRCP [25,26].

Quantitative MRCP (MRCP+) has been developed to address these gaps. It provides an accurate quantitative assessment of the biliary anatomy from MRCP images using artificial intelligence (AI) algorithms [27,28,29]. MRCP+ has shown early clinical utility to support diagnosis, risk stratification and prediction of outcomes in patients with PSC [28,30,31,32,33] including in the evaluation of benign vs. malignant biliary obstructions [34] as well as improving the assessment of High Grade strictures [35]. Furthermore, in addition to showing utility in the objective long-term monitoring of patients [36], MRCP+ has shown utility in identifying high risk patients [29,33] and predicting clinical outcomes including hepatic decompensation, biliary complications, liver transplantation and death [37,38,39]. To this effect, MRCP+ has been included in the recent European Association for the Study of the Liver (EASL) on sclerosing cholangitis as a “prognostic tool for prediction of outcomes in PSC” [40]. Thus, adopting MRCP+ quantitative metrics in clinical trials and clinical practice can potentially improve treatment options, risk stratification, and surveillance of patients with PSC.

As MRCP+ is not routinely used in clinical settings, barriers to its clinical adoption still need to be explored. When assessing the adoptability of a new technology, evaluation of the user’s perceived usefulness (PU) of the technology in their setting is key [41]. PU is linked to technology adoption [42] as it defines the degree to which users believe a particular technology, and its features will help them achieve their goals (precision medicine). PU has been used to evaluate perceptions and acceptability of technologies in a range of contexts within the healthcare industry including the evaluation of health practitioners’ views on electronic technology adoption [43], mobile health technology acceptance [44], and technology acceptance in palliative care [45]. This pilot feasibility study aimed to explore stakeholder perceptions by evaluating the potential utility, value, and practicality of using MRCP+ to manage PSC in secondary care in NHS England alongside current barriers to its adoption in clinical trials and practice. Although there is a growing body of evidence which shows the clinical utility of MRCP+ as part of the PSC clinical management pathway, we sought to understand the potential impact and feasibility adoption would have on patient management.

## 2. Methods and Materials

Early economic evaluation to evaluate the perceived usefulness and acceptability of using MRCP+ to support diagnosis, monitoring and management of patients with PSC was performed independently by Health Innovation Oxford (previously Oxford Academic Health Science Network (AHSN)). The Oxford AHSN (now known as Health Innovation Oxford) is one of fifteen regional networks created by NHS England in 2013 to fast-track effective health and care innovations into everyday practice. Based at Oxford University Hospitals NHS Foundation Trust and Oxford Health NHS Foundation Trust, it receives core funding from NHS England, supplemented by contributions from local trusts and Integrated Care Systems. By convening NHS organisations, academic institutions, local authorities, industry partners, and the voluntary sector, Health Innovation Oxford identifies promising new diagnostics, digital solutions, care models, and medical devices. It then provides the project management, evaluation tools, training, and stakeholder engagement needed to pilot these innovations, assess their clinical and economic impact, and scale them across the NHS. All activities are carried out in close collaboration with commissioners and providers to ensure alignment with national standards for quality, safety, and procurement.

This study was part of a project to examine the impact of quantitative MRCP scans on clinical decision making in adult patients with PSC (NCT04015310). This study was funded by an Innovate UK grant (award number: 30166) aimed at supporting early economic evaluation of innovative medical technologies and had local ethical approval from the West Midlands–Edgbaston Research Ethics Committee (reference: 19/WM/0326).

### 2.1. Standard Care Pathways

**Figure 1:**
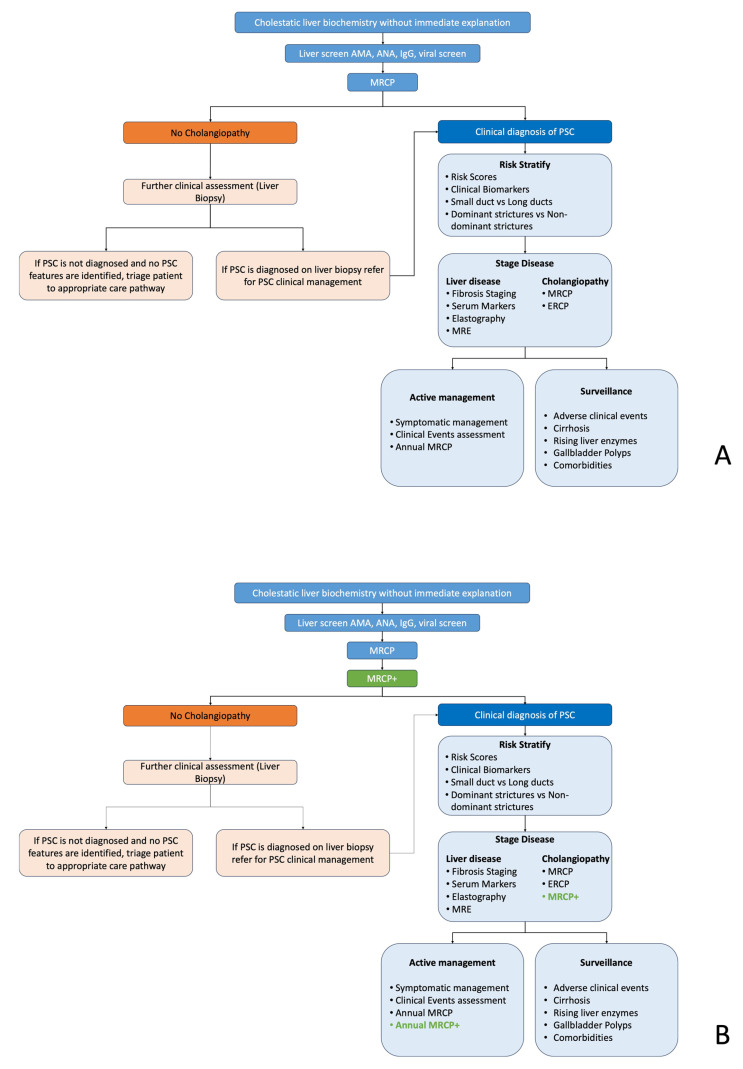
Schematic diagram of the standard of PSC adapted from UK and European clinical guidelines. (**A**) Current pathway; (**B**) proposed pathway incorporating quantitative MRCP. AMAs: antimitochondrial antibodies, ANAs: antinuclear antibodies, IgG: immunoglobulin G, MRCP: magnetic resonance cholangiopancreatography, MRCP+: quantitative magnetic resonance cholangiopancreatography, PSC: primary sclerosing cholangitis and ERCP: endoscopic retrograde cholangiopancreatography.

Following initial screening, as part of the standard of care (SoC) patient pathway (Figure 1), patients with suspected PSC receive an MRCP to support stratification to rule-in or rule-out disease. Patients without any signs of cholangiopathy are excluded from further investigations; those with questionable disease/where a clear PSC diagnosis cannot be obtained (or have potential comorbid disease) are re-routed for further clinical assessment (liver biopsy) if suspicious pathology is present. Those with evidence of PSC are risk stratified depending on disease severity and managed according to site SoC. In the event of severe disease, patients typically undergo endoscopic retrograde cholangiopancreatography (ERCP).

The disease progression in these patients is continuously actively monitored using risk scores, liver enzymes, and imaging (Figure 1) to support early detection of the development of clinical events (cholangitis, dominant strictures, gallbladder polyps, cirrhosis, cancer, etc.). Clinical guidelines recommend that patients with PSC undergo annual MRCP alongside continuous symptom and clinical evaluation to better monitor subclinical disease progression. Ursodeoxycholic acid (UDCA) has been used to help prevent or delay liver damage in PSC [40]. However, evidence shows that while it does improve liver biochemistry, it does not improve patient outcomes and can be harmful if provided in high doses [40]. Although the only curative intervention for PSC is liver transplantation, de novo disease develops in 10–40% of cases [40].

### 2.2. Feasibility Assessment and Stakeholder Identification

The feasibility of implementing MRCP+ as a healthcare technology in care pathways was investigated. The Lean Assessment Process (LAP) methodology [46,47] was used to assess the potential impact and feasibility of adopting MRCP+ in the PSC care pathway within the NHS. As the LAP methodology provides a structured process for identifying key stakeholder views that might support the prioritisation of clinical needs and alignment of stakeholder preferences, the impact that the SoC pathway, including MRCP+, would have on PSC management was evaluated. As part of this assessment, price as a barrier to adoption was investigated. In this study, price as a barrier to adoption was defined as the degree to which a person believes that the price of a system does not outweigh its clinical benefit and warrants its adoption into the national healthcare system. In healthcare, when assessing the usefulness of a technology, alongside the logistical assessment of barriers to adoption, such as labour force, local labour market and skills gaps, evaluation of the product/technology price point plays a key role in assessing technology uptake.

Alongside a detailed literature review and systematic evaluation of UK and European clinical guidelines (BSG, UK-PSC, European Association for the Study of the Liver [EASL]), semi-structured interviews with key stakeholders in the PSC management and care (fourteen hepatologists and two patient representatives) were conducted. A structured questionnaire regarding the current and proposed pathways along with scoring questions on product perceptions of MRCP+ made up the semi-structured interviews with a 7-point Likert scale used to record all answers. All included hepatologists were working in NHS England with expert experience (>10 years) in the clinical management of patients with PSC and in developing clinical pathways for PSC/hepatobiliary disease. Participating hepatologists were from the following NHS trusts: Leeds Teaching Hospitals, Royal Berkshire NHS Trust, Cambridge University Hospitals, University College London Hospitals, Oxford University Hospitals, Milton Keynes University Hospitals, Kings College Hospital NHS Foundation Trust, Nottingham University Hospitals and Great Western Hospitals. Patient representatives (who were also patients with PSC) were from the PSC Support (UK PSC Charity). Participants did not receive financial compensation for participating in this study.

### 2.3. Lean Assessment Process Methodology

The LAP comprises a mixed-methods evaluation of operational maturity across five core domains: value definition, process flow, pull (demand-driven activity), continuous improvement, and leadership engagement. Semi-structured interviews are conducted using an interview guide (non-validated) that combines open-ended questions with 7-point Likert-scale items aligned to each domain. Interview recordings are transcribed verbatim, and transcripts are coded against a pre-defined codebook structured around the five lean pillars. Qualitative data are subjected to thematic analysis, with individual codes aggregated into higher-order themes (e.g., bottleneck identification, standard work adoption, and feedback loops). Concurrently, quantitative maturity scores are generated by converting Likert responses into domain-level metrics. Coding discrepancies are reconciled through consensus review, and emergent themes are validated by cross-referencing with quantitative scores to ensure integrated interpretive validity. The resulting output is a lean-maturity profile that highlights common barriers and pinpoints high-impact opportunities for process improvement [46,47].

Interviews and data collection were carried out independently by the Oxford AHSN, which selected participants and conducted all sessions. Manufacturer affiliates remained blinded to the identities of the clinicians and had no access to raw interview recordings, transcripts, or any other identifiable collected participant data.

### 2.4. Methodology and Research Hypothesis

Perceived usefulness (PU) has been identified as one of the primary factors used to predict the usage of technology in various settings. In this study, from the perspective of healthcare technology, PU was defined as the degree to which a person considers that using MRCP+ as part of management will be advantageous to them. In other words, if clinicians/patients feel/think that MRCP+ is useful and will improve their understanding of disease severity and enhance their work performance (by impacting clinical decision making) this perceived usefulness will encourage them to use the technology. We hypothesised that PU has a significant effect on the intention to use MRCP+ as part of patient management. To support evaluation of PU, two key aspects were investigated:

- Ease of Use (EoU)—the degree to which a person believes that using a system would be free of effort plays an important role in the usage and adoption of a technology. When users perceive a technology to have a positive impact on their tasks and activities, they will have the desire to use it as they will find it convenient and of (clinical) benefit.
- Trust (T)—the degree to which a person is willing to believe that their expectations will be met when using a particular system. Studies evaluating the usefulness and implementation of technologies have shown that the level of trust users have in a new technology can significantly impact their desire to use it.

All data were collected between 2020 and 2021. The clinical pathway shown in Figure 1 (including routes and assumed inclusion of MRCP+) was developed from clinical guidelines and discussion with experienced hepatologists and PSC experts. Quantitative structured questionnaires were used to collect the responses from stakeholders using a 7-point Likert scale. Development of the questionnaires followed the following steps:

1. Technology definition: undertaken by the manufacturer in collaboration with the AHSN.
2. Questionnaire design: AHSN developed a questionnaire that including statements related to PU including the extent to which users perceive the technology as useful in achieving their goals or fulfilling their needs. A 7-point Likert scale was used to measure responses (e.g., strongly agree, agree, neutral, disagree, strongly disagree).
3. Pilot testing: A pilot test of the questionnaire with an individual from the manufacturer and another from the AHSN was performed to identify any potential issues with the questionnaire’s clarity, wording, or response options.
4. Questionnaire administration and data collection: 16 interview questions were circulated to the stakeholders, and semi-structured recorded interviews were conducted.

The questionnaire was divided into two sections to cover the main elements for the development of the proposed hypotheses. Once the data were collected, the mean score for each statement/question was computed. The medians of the median scores for the questions pertaining to the ease of utility were taken as representing the perceived ease of use score based on the participants’ responses. This median score was then represented as a percentage reflecting the perceived ease of use. Similarly, the medians of the median scores for the questions pertaining to trust associated with using MRCP+ were taken as representing the perceived ease of use based on the participants’ responses. For both metrics, higher percentages were taken to indicate higher perceived ease of use/trust, while lower percentages suggest lower perceived ease of use/trust. Overall PU was calculated as a function of ease of use and trust (i.e., PU = *f* [EoU + T]). As liver biopsy is the current reference standard for disease assessment, in this study, a good PU which satisfies our hypothesis was defined as having a perceived score greater or equal to that of liver biopsy. Cronbach’s alpha (CA) was used to assess reliability with a CA > 0.70 interpreted as being reliable. All statistical analyses were performed using R.

## 3. Results

### 3.1. Questionnaire Results

**Figure 2:**
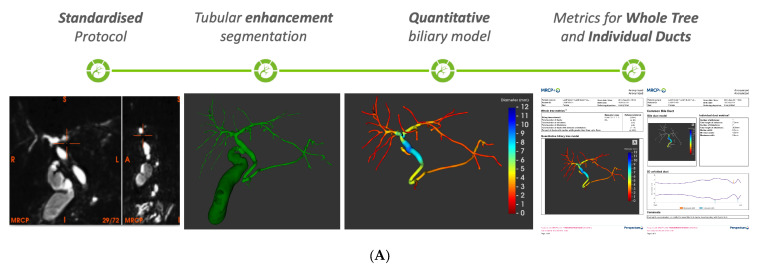

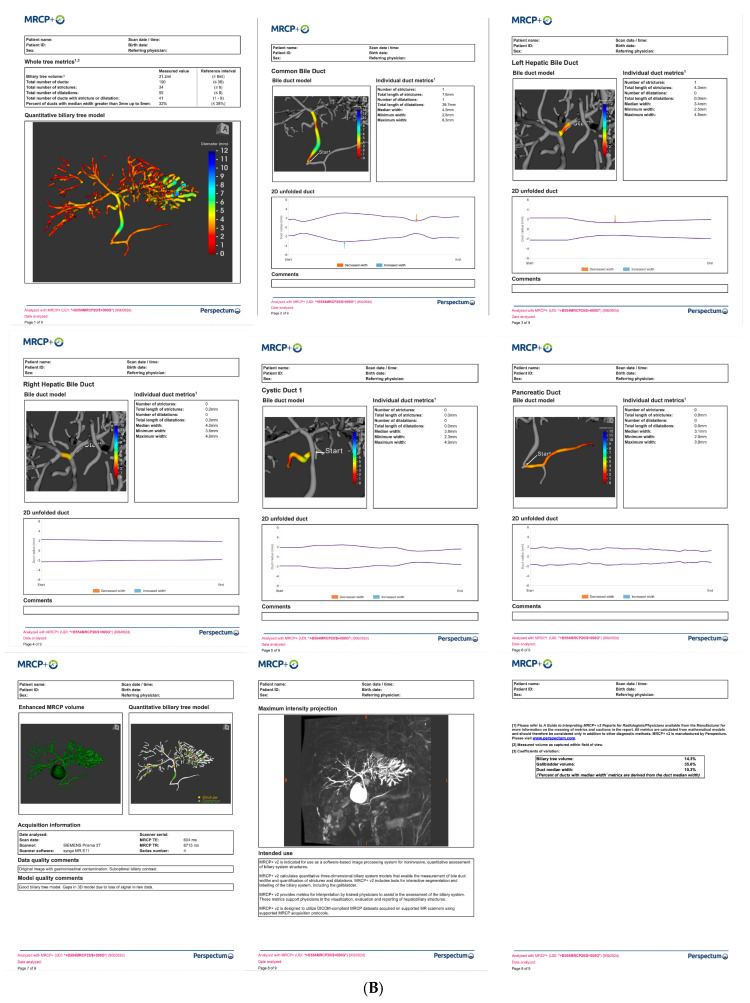
Illustration of (**A**) the quantitative imaging of the biliary tree showing the comprehensive assessment of the pancreatobiliary tract morphology and the resultant quantitative magnetic resonance cholangiopancreatography (MRCP+) report and (**B**) an MRCP+ report. The MRCP+ report gives a quantitative summary of biliary morphology and 2D graph(s) of unfolded ducts of concern to enabling easier monitoring of stricture(s).

Hepatologists (clinicians) and PSC Support patient representatives (patients) were asked to complete a structured questionnaire and semi-structured interview. Data were collected over a 12-month period. A total of 16 responders (14 clinicians and 2 patients) were received. Table 1 and Table 2 shows the score for each statement/question. Consensus points concerning standard of care as well as those concerning the use of quantitative MRCP+ as part of standard of care are shown in Supplementary Tables S1 and S2, respectively. All individual responses were kept confidential by the AHSN; Supplementary Figure S1 shows the discussion guide used during the interviews. An illustration of the quantitative imaging of the biliary tree showing the comprehensive assessment of the pancreatobiliary tract morphology and the resultant quantitative magnetic resonance cholangiopancreatography (MRCP+) report is shown in Figure 2. In addition, Figure 2B shows an illustration of the industry standard MRCP+ report. Contained within the report are the biliary tree model alongside a summary of whole tree metrics (page 1), quantitative model of the common bile duct and 2D unfolded duct (page 2), left (page 3) and right (page 4) hepatic bile duct models and 2D unfolding, cystic (page 5) and pancreatic (page 6) duct models and 2D unfolding, quantitative model highlighting strictures and dilatations only (page 7), MRCP maximum intensity projection (page 8), and volumetry summary (biliary tree and gallbladder) alongside manufacturer interpretation guide (page 9).

### 3.2. Baseline Evaluation of Current Landscape

Firstly, to obtain a baseline understanding of the usefulness of existing technologies to support diagnosis, responders were asked to score their perceptions on currently available technologies (MRCP, ERCP and liver biopsy) (Table 1). Traditionally read MRCP scored higher than liver biopsy (68%) and ERCP (50%) due to its noninvasive nature. Although MRCP was noted as the gold standard for diagnosis (as also noted in clinical guidelines), interobserver variability, the lack of expert radiologist, and the lack of resolution to see more granular aspects of disease such as intrahepatic ducts were noted as disadvantages (Table 1).

When asked about the potential diagnostic and monitoring options which will potentially be available in the near future (within 3–5 years), responders noted that PSC is an area with strong development potential. This was especially true as there are numerous late phase clinical pharmacotherapy trials but a lack of regulatory approved surrogate endpoints to validate the efficacy of new and experimental therapies (Supplementary Table S1). Similarly, when the potential for (new) biomarkers to guide treatment choices and monitor status/progression of PSC was questioned, responders noted the high prevalence (15%) of bile duct cancers which develop in this patient population resulting from poor-to-suboptimal screening and surveillance strategies.

### 3.3. Feasibility Assessment

The LAP methodology promotes an iterative and data-driven approach to technology assessment, enabling organizations to make informed decisions and reduce the likelihood of investing in infeasible or high-risk technologies. As part of the LAP assessment, the feasibility of implementation, potential impact of adoption and impact on SoC were investigated.

When asked to comment on the current patient pathway (diagnosis and management), alongside reporting that their practice aligns most with BSG and UK-PSC [1] and EASL guidelines [40], clinicians mentioned the unmet needs in the management of PSC (Supplementary Table S1). Chief amongst the unmet needs reported was the lack of quantitative objective markers to support management (early detection, monitoring, therapeutic response, and prognosis).

When asked to comment on the potential impact and feasibility of adopting MRCP+, clinicians noted that like other imaging technologies, such as computerised tomography (CT) and positron emission tomography (PET), tertiary specialist hepatology centres would likely be the first to implement such technologies. For instance, tertiary centres are likely to show interest and make local business cases to clinical commissioning groups (CCGs) for commissioning the technology as they have the expertise and capabilities to accommodate new technologies. Limited MR capacity was noted as a factor which negatively impacts the adoption of MRCP; however, as it is already widely used in the management of patients with cholestatic disease, this was not viewed as a barrier to adoption (Supplementary Table S2).

Current clinical care pathways make use of a range of procedures and techniques (liver biopsy costs GBP 726 (without including the cost of complication), ERCP costs GBP 435, Percutaneous transhepatic biliary drainage costs GBP 1206, and cholangioscopy costs GBP 2810 for visual inspection only) in the diagnosis of PSC [48]. Thus, as part of the interview, stakeholders were asked to provide their views in relation to price of MRCP+ as a barrier to adoption. There was good internal consistency between responders on the relationship between price-point and the use of the technology to support diagnosis as well as frequency of use for monitoring. For diagnosis support, a price-point ranging between GBP 100 to GBP 500 was noted as unlikely to affect management with good consistency (Cronbach’s alpha: 0.836 [95% CI: 0.552–0.930]). Although responders noted that higher price points may make clinicians more reluctant to tests, there was excellent consistency that if used annually (at a similar frequency to MRCP), a price-point ranging between GBP 100 to GBP 500 was unlikely to affect management (Cronbach’s alpha: 0.904 [95% CI: 0.676–0.962]).

The impact that the SoC pathway, including MRCP+, would have on PSC management was deemed as being significant, as clinicians noted that following successful adoption into tertiary (academic) referral centres, as evidence of clinical utility grows, the use of MRCP+ will likely spread to district general hospitals. Although the use of MRCP+ was noted to likely be tariff-based using referrals with variable likelihood for the use of block contracts, the future adoption of MRCP+ within NHS England was viewed to likely follow that used by other similar imaging modalities (CT and PET).

### 3.4. Perceived Usefulness

**Figure 3:**
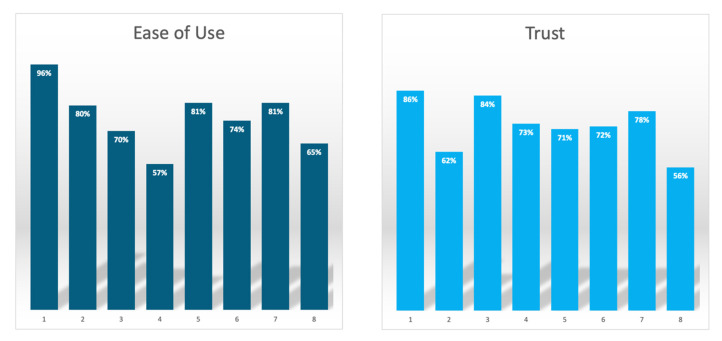
Mean scores for the perceptions around the ease of use and trust of using quantitative magnetic resonance cholangiopancreatography (MRCP) making up perceived usefulness.

The perceived ease of use for MRCP+ had a mean score of 76% whilst the perceived trust had a mean score of 73% (Table 2, Figure 3). This was higher than the mean perceptive scores for both the use of liver biopsy and ERCP (Table 2). The overall perceived usefulness for the use of using MRCP+ as part of patient management was 74%. When compared to the perceptions of using liver biopsy (68%), the PU relating to the use of MRCP+ as part of PSC management was higher; therefore, the hypothesis was accepted.

## 4. Discussion

In this pilot feasibility assessment evaluating the feasibility and perceived usefulness of introducing quantitative MRCP into the care pathway of patients with PSC as an objective tool, we identified three key findings. Firstly, quantitative MRCP was found to have good perceived ease of use and perceived trust amongst clinicians and patient advocates, which resulted in its good perceived usefulness (greater than that of both ERCP and liver biopsy). Secondly, quantitative MRCP was found to have the capability to address some unmet needs in the management of PSC as responders perceived the technology to have the ability to objectively characterise the biliary tree producing metrics which can support multiple facets of disease management (early detection, monitoring, therapeutic response, and prognosis). Third, in relation to price as a barrier to adoption, for both diagnosis and long-term monitoring support, a price point ranging between GBP 100 to GBP 500 was noted as unlikely to affect the technologies use for supporting patient management.

Quantitative MRCP is a validated image-processing tool that provides quantitative metrics of the biliary tree, providing quantitative measures that facilitate assessment of ductal anatomy, using a standardised imaging protocol and image processing software. MRCP+ uses the same non-contrast 3D MRCP acquisition as standard MRCP but also increases the resolution of the acquired images with isotropic acquisition. In conjunction with a dedicated post-processing method which enhances the visualisation of the biliary tree, suppressing the low intensity signal abdominal structures, MRCP+ improves the visualisation of small calibre ducts with automatic and quantification measurements that provide ducts diameter, number of ducts, number of strictures, number of dilatations and biliary tree volume, among other biliary metrics [27,49]. Therefore, by using an AI-driven tubular enhancement imaging protocol which suppresses gastrointestinal (gastric/duodenal) contamination, MRCP+ protocols avoid the need to use intramuscular glucagon administration, which may be associated with undesirable side-effects, to improve final image quality. Thus, when compared to traditional MRCP, which relies on subjective assessment, MRCP+ is more objective with good technical (repeatability and reproducibility) performance [49].

To obtain a comprehensive view of the impacts that the inclusion of MRCP+ would have on the patient pathway, clinicians representing different sizes of academic, referral and non-academic NHS Hospital Trusts across England were included. Furthermore, to obtain a wholistic view, patient representatives from a UK PSC Charity (PSC Support) were also included as participants in the study. All participating clinicians follow British and European clinical guidance for the management of PSC and noted the lack of quantitative objective markers to support management as an unmet clinical need. Quantitative MRCP has been noted in the EASL clinical guidelines as having potential prognostic utility in the prediction of outcomes in PSC [40]. Regarding clinical utility, studies in the literature have shown the ability of quantitative MRCP metrics to support diagnosis, risk stratification [29,31] and prediction of outcomes in PSC [33,34,37,38,39]. Moreover, studies comparing MRCP+ to traditional MRCP [35] have shown that in addition to positively impacting decision making, MRCP+ reduced inter-reader variability (even amongst expert readers), indicating its potential to support standardized MRCP assessment and subsequent management for patients with PSC. Furthermore, when evaluating evidence specifically pertaining to risk prediction, MRCP+ has been shown to outperform the MAYO [39] and ANALI [38] risk scores in head-to-head comparison. Thus, the growing body of evidence reporting the utility of MRCP highlights the clinical utility this technology can offer, and the support the metrics could give to the monitoring of therapeutic pharmacotherapies (especially as there are no current objective tools to assess the changes in the biliary tree in response to treatment) [36]. Capacity has been noted as a limiting factor hindering the uptake of MRI based technologies; however, as MRCP is already used as a first-line noninvasive tool in the management of PSC, the addition of MRCP+ was not viewed as a barrier to adoption.

Evaluation of the ease of use and trust in using quantitative MRCP to support PSC patient management showed a good agreement between all participating clinicians. Specifically, quantitative MRCP had a higher perceived usefulness when compared to the use of liver biopsy and ERCP. This was primarily driven by the noninvasive nature of the technology, but clinicians also noted the non-subjective nature of the test as a significant contributor. This is important as traditional MRCP is subjective and thus suffers from high inter-observer variability which may affect non-academic centres more (due to the scarcity of expert radiologists).

In addition to ease of use and trust, price can be a significant barrier to adoption for new technologies within the NHS. As part of this study, participants were asked about the impact that a price ranging between GBP 100 to GBP 500 would have on the use of the technology to support diagnosis as well as frequency of use for monitoring. Findings showed that a price of up to GBP 500 was acceptable and as this cost was unlikely to affect management. Responses showed good-to-excellent internal consistency with Cronbach’s alpha of 0.836 for diagnostic support and 0.904 for annual monitoring. Conventionally, Cronbach’s alpha values ≥ 0.8 are considered to indicate good reliability and ≥0.9 excellent reliability. In this study, wide 95% confidence intervals around Cronbach’s alpha were observed for the evaluation of MRCP+ as part of diagnostic support, which is most likely driven by the study’s small sample size and the limited number of items assessing the criterion. Future studies should include larger groups of clinicians, and where possible additional items per construct. This will inherently increase the precision of reliability estimates (yielding narrower confidence intervals), permit more robust item-level and subgroup analyses, and improve the generalizability and interpretability of the internal-consistency measures.

Clinicians also suggested that similar to other imaging technologies such as CT and PET, tertiary specialist hepatology centres would likely be the first to implement such technologies as they have the expertise and capabilities to accommodate them. There was consensus among clinicians that subsequent adoption by general hospitals was likely to follow successful adoption into tertiary (academic) referral centres.

Successful integration of any new technology into healthcare systems depends on multiple domains beyond stakeholder perceptions alone. In addition to assessing price as a barrier, formal health-economic evaluations, such as cost-effectiveness analyses or budget-impact models, are required to demonstrate that a technology delivers value for money. Equally important is the analysis of resource utilisation, particularly in time-constrained systems like the NHS. While the focus of this study was to ascertain stakeholder perceptions relating to MRCP+, future studies should look to collect these real-world data needed for adoption decisions. To date, MRCP+ has achieved reimbursement under Category III CPT codes 0723T and 0724T in the United States and is already in clinical use to support cholangiopathy assessment, including in PSC patients. However, its system-wide feasibility and cost-effectiveness within the NHS will require dedicated implementation studies with larger, representative samples and formal economic modelling. These models should also evaluate the impact on resource use and the return on investment associated with the use of MRCP+.

There were some strengths and limitations to this study. Firstly, there is no consensus on how best to report perceptions of new medical technologies which have not been formally adopted into the NHS medical device framework. Perceived usefulness is a measure that typically contributes to the Technology Acceptance Model (TAM), one of the most widely used extensions of the Ajzen and Fishbein’s Theory of Reasoned Action (TRA). In this study, we did not evaluate the TAM as quantitative MRCP is not currently used in the NHS, and hence an analysis evaluating the drivers of perceived usefulness was not applicable. Nevertheless, early evaluations of the perceived ease of use and perceived trust were conducted and compared to the perceived usefulness of existing techniques currently used in patient care. Although the majority of questions required clinical knowledge of patient management and thus may not have been applicable to the patient advocates, precision medicine is patient centric in nature, and thus it is important to also evaluate patients’ perceptions to the use of new technologies to support their management. In routine practice, as well as noted in clinical guidelines, principal clinical responsibility for diagnosis, longitudinal management, surveillance (including cholangiographic assessment, cancer surveillance and transplant referral), and decisions about endoscopic and medical interventions sits with hepatologists in specialist hepatology centres and clinics. Therefore, in our study, we focused primarily on understanding the perceived utility of MRCP+ from this specialist group. Nevertheless, like most chronic liver diseases, PSC management is multidisciplinary and thus, views from all involved stakeholders should be considered. Thus, future studies should look at inclusion of a larger sample of diverse healthcare professionals (including radiologists, hepatobiliary surgeons, transplant surgeons, specialist nurses, etc.), NHS budget holders and other stakeholders to validate the results presented herein. Assessing patients’ views is essential when evaluating a technology’s perceived usefulness as their perspectives also contribute to determining acceptability, usability, and the likelihood of real-world uptake. In this study, only two patient representatives (both living with PSC) contributed to our work. Although this was beneficial as their input was informative and improved the acceptability of study materials, we acknowledge that this limited representation may constrain generalizability. Future studies should engage broader patient groups and professional patient societies to more broadly assess patient perceptions and acceptance of technologies proposed for inclusion as part of their care. These studies should not only assess attitudes toward novel technologies but also evaluate perceptions of existing care pathways to ensure innovations address gaps that patients themselves identify.

This study serves as part of the basis for further research on the perceptions of new medical technologies to support patient management as this is an area requiring further development. With the availability of more clinical data addressing current unmet needs, adoption studies evaluating the TAM, and in particular the implications of the use of the technology within the broad patient management landscape, will allow for the generation of unique verbiage that captures the rationale for adopting such technologies within patient standard of care. In this study, we intentionally compared MRCP+ against liver biopsy and ERCP because these invasive procedures remain the reference standards for histological grading of cholangitis, detection of overlapping autoimmune features, confirmation of ductal anatomy, and for delivering therapeutic interventions. However, we did not perform a direct comparison between quantitative MRCP+ and conventional MRCP as the primary aim was to investigate and understand the perceived usefulness (ease of use and trust) as well as exploring stakeholder perceptions around the potential clinical utility, value, and practicality of using MRCP+ to manage PSC. Given that current clinical guidelines recommend MRCP as the first-line imaging modality for PSC diagnosis and surveillance, a head-to-head evaluation of standard MRCP versus MRCP+, ideally involving general and specialist radiologists, would yield critical insights into real-world adoption. Accordingly, future work should incorporate both clinical validation and direct comparisons of these two MRCP techniques. However, it is important to note that, echoing current trends in clinical digital technologies (for example, digital pathology), MRCP+ is likely to act as a complementary tool that enhances MRCP interpretation and enables more objective assessment. This is because digital methods offer inherently more repeatable and reproducible measurements than human interpretation, which is subject to greater variability. Lastly, although the survey data were collected in 2020–2021, they fall within the timeframe of prevailing guidance (EASL PSC update, 2022; BSG/UK-PSC guideline, 2019). Clinical management of PSC and the central role of MRCP have not changed substantively since that period, so clinician views on MRCP+ (usability, trust, and price sensitivity) are unlikely to have shifted materially. We therefore consider these perceptions relevant.

## 5. Conclusions

In conclusion, our findings show that there is strong clinical interest amongst hepatologists in using quantitative MRCP in the PSC management pathway, particularly for diagnosis and monitoring. The quantitative and objective assessment of patients’ MRCPs may facilitate less subjective and more definitive means to monitor patient progression than is currently available. This non-invasive approach has the potential to address many unmet needs in the PSC pathway, including reducing the subjectivity associated with the current qualitative MRCP, and enabling objective, trackable and repeatable measurement of duct diameter throughout the biliary tree.

## Figures and Tables

**Table 1:** Baseline evaluation of current landscape. Mean score assessing the usefulness of existing technologies to support diagnosis and monitoring in PSC. MRCP: magnetic resonance cholangiopancreatography; ERCP: endoscopic retrograde cholangiopancreatography.

Techniques	Mean Score	Pros	Cons
MRCP	81%	Non-invasive and can spot changes in the bile duct. Preferred noninvasive route for diagnosis of PSC.	Reporting is subjective; therefore, smaller centres with less-experienced radiologists may be under- or over-interpreting images. There is no quantification of the biliary tree. Lacks the resolution to see intrahepatic duct.
ERCP	50%	Enhanced diagnostic tool. Provides a good 3D view of the cholangiogram.	Rarely used for diagnosis in the UK to treat PSC patients. Only in therapy for strictures. Invasive with high risk of complications.
Liver biopsy	68%	Considered to be more accurate than MRCP and ERCP as It shows the liver pathology.	Subject to sampling error. Small portion of liver is analysed. Risk of complications and mortality.

**Table 2:** Mean scores for the perceptions around the ease of use and trust of using quantitative magnetic resonance cholangiopancreatography (MRCP). PSC: primary sclerosing cholangitis.

Assessment of Perceptions of Using Quantitative MRCP	Mean Score
**Perceived ease of use**	
Quantitative MRCP is a noninvasive diagnostic technique	96%
Quantitative MRCP facilitates the reporting of whole biliary tree metrics	80%
Quantitative MRCP suppresses noise and provides quantitative, visually rich models of the biliary tree from routine 3D MRCP	70%
Quantitative MRCP reports report whole tree metrics, such as duct number, biliary tree volume and gallbladder volume	57%
Quantitative MRCP reports report single duct metrics such a stricture and dilation length and number	81%
Quantitative MRCP allows the production of 2D graph of unfolded ducts, enabling easier monitoring of stricture(s)	74%
Quantitative MRCP allows for comprehensive assessment of the pancreaticobiliary tract morphology	81%
Quantitative MRCP allows for 3D visualisation which can aid improved characterisation of gallstones	65%
**Perceived trust**	
Quantitative MRCP is an accurate means for the early detection of PSC	86%
Quantitative MRCP is a way to accurately determine the stage of PSC which can support a prognosis	62%
Quantitative MRCP allows objective assessment of the biliary tract for monitoring of PSC progression	84%
Quantitative MRCP allows improved diagnosis and monitoring of PSC	73%
Quantitative MRCP is a clinically meaningful way to quantitatively define dominant bile stenosis	71%
Quantitative MRCP allows for enhanced visualisation of the biliary tree to support pre-surgery planning	72%
Quantitative MRCP is an effective tool for assessment of the biliary system pre- and post-liver transplant	78%
Quantitative MRCP is an accurate way to detect the early stages of cholangiocarcinoma	56%

## Data Availability

The data and analytic methods used in this study remain the property of the study sponsors. All deidentified participant data may be made available to other researchers upon request following permission, investigator support and following a signed data access agreement.

## Supplementary Materials

The following supporting information can be downloaded at: https://www.mdpi.com/article/10.3390/healthcare13202630/s1. Figure S1: The discussion guide used to collect stakeholder insights; Table S1: Consensus points concerning standard of care raised by clinicians; Table S2: Consensus points concerning the use of quantitative MRCP+ as part of standard of care raised by clinicians.
