# Supplementary material for: Quantitative MRCP as Part of Primary Sclerosing Cholangitis Standard of Care in the National Health Service in England: A Feasibility Assessment Among Hepatologists
Source: Healthcare (Basel). 2025 Oct 20;13(20):2630. doi: 10.3390/healthcare13202630 (PMC12562387; doi:10.3390/healthcare13202630)
Supplement: Supplementary file 1 [file healthcare-13-02630-s001.zip › supplementary files/Supplementary figure S1.pdf]

Supplementary figure S1: The discussion guide used to collect stakeholder insights

Oxford Academic Health Science Network

STRATEGIC AND INDUSTRY PARTNERSHIPS

(The current guide has been developed for clinicians. It is to be modified as appropriate for other stakeholders, e.g. nurses, patients, commissioners, etc.)

Background

Oxford Academic Health Science Network (Oxford AHSN) is a partner in a study focused on monitoring patients with primary sclerosing cholangitis (PSC). We are working with partners towards the objective assessment of introducing a new product (quantitative MRCP [MRCP+]) into the PSC care pathway. In support of this project we would like to discuss how you currently manage patients with PSC and gain your views on the potential utility that the introduction of MRCP+ may offer in the immediate and on-going management of patients with PSC.

1) Current Landscape

a. Firstly, we would like to explore the current standard of care based around how you would manage patients diagnosed with PSC.

Please could you describe how you currently manage patients with PSC and insight into options for monitoring and treating patients?

We shared a schematic diagram in the pre-read (Figure 1) that outlines the care pathway for patients with PSC.

b. Please could you advise how well you consider this might represent the current care pathway?

[Probe the monitoring – timings, approaches and degree of satisfaction with current approaches]

c. Please could you outline the (**any**) relevant (published) care pathway(s) you consider might be applied in managing patients with PSC?

d. Please outline:
   i. How you would diagnose PSC

[Probe what is used as indicative and what definitive?]

   ii. How treatments, such as UDCA, are used to treat patients with PSC?
   iii. How frequently patients are reviewed, which tests might be routinely applied and at what intervals?

e. Are there any relevant guidelines for managing patients with PSC and which you apply in your practice: For example:
   i. AASLD/EASL guidelines (and any specific pieces of guidance)
   ii. Local guidelines
   iii. Royal College guidelines
   iv. Others - please specify

Figure 1. Current care pathway for PSC

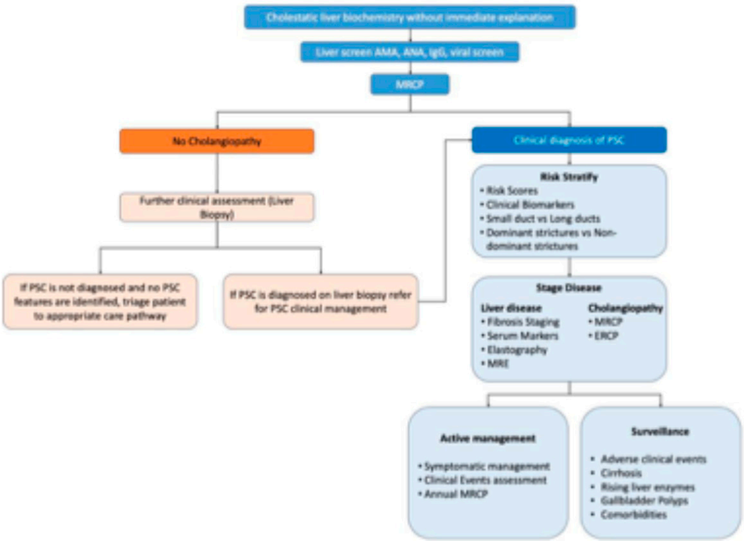

f. In the context of PSC management could you:
   i. Outline the key concerns you have in the management of PSC patients?
   ii. Comment on how well the service currently meets the needs of PSC patients?
   iii. Comment on the level of unmet need in monitoring PSC patients, specifically in the context of identifying those PSC patients
      - Who might need better management of their immunosuppression
      - And/or those who are close to requiring a liver transplant?
      - Who might have sub-optimal management of their PSC which impacts on their quality of life

2) Future trends in the management of PSC

Please could you comment on:

a. Your understanding of the current trends in the incidence and prevalence of PSC in your area
b. The potential future treatment, diagnostic and monitoring options of which might become available in the next 3 to 5 years

[Probe (as appropriate) around how these might change the care pathway and decision point in monitoring]

c. The potential for increased use of diagnostic tests/biomarkers to guide treatment choices and monitor status/progression

3) Technologies currently used in the monitoring and management of PSC

Please could you comment on:

a. The treatment regimens of choice for the management of PSC patients
b. The monitoring regimens routinely used in the management of hepatitis patients

[Probe whether there are standard (SOC) therapies and diagnostics and whether there is much variation around this (clinician choice)]

c. What are your views on the use of liver biopsies for management support of patients with PSC? When would you use it?
d. What are your views on the use of blood tests for monitoring patients with PSC?

4) MRCP+ Product assessment

The pre-read included details of how MRCP+ can be used in the monitoring of patients with PSC. We are now going to ask questions about the potential utility of this approach to monitoring and its utility in the management of patients with PSC.

[Do you have any questions or need clarification of the way the MRCP+ provides data on the state of the biliary tree in PSC patients? This is covered in the pre-read document]

MRCP+ is:

- A non-invasive MRI-based technology that has gained FDA 510(k) clearance and CE marking to aid clinicians in the assessment and monitoring of cholangiopathy.
- A diagnostic and monitoring aid for PSC that uses standard noncontrast MRCP to model the biliary tree.
- Non-invasive and models the biliary tree providing quantitative measures of ducts strictures, dilatations and volumes of biliary structures.
- Is delivered as a service and does not require any additional hardware installation and is standardised across main manufacturers and field strengths.

MRCP+ uses images acquired from standard clinical MRI scanners to model the biliary tree to:

- Highlight the presence of strictures and dilatations,
- Increase the resolution of the acquired images and improves the visualisation of small caliber ducts, enabling better assessment

MRCP+ metrics have been shown:

- To be correlated with histological scores and with commonly used risk scores
- Demonstrate utility in stratifying patients with fibro-inflammatory liver disease
- To be strongly predictive of clinical outcomes

5) MRCP+ Product perceptions

a. Based on the way MRCP+ works and the potential to provide quantitative metrics regarding biliary structures please could you provide your thoughts on the utility of the test in the management of patients with PSC?

[Probe where test may offer most utility and where might be alternative to other forms of testing]

b. Do you think the test could be adopted for use into the PSC care pathway?

c. Based on the pre-read schematic of the care pathway (Figure 1) we would like your thoughts about the potential places where the MRCP+ might be used for monitoring (and diagnosis). Please could you share your opinion against the potential places where MRCP+ might be used.

d. Please describe the advantages you perceive for MRCP+?

e. Please highlight the product characteristics that you consider might impact adoption (positively and negatively)

f. What would you say were the key parameters the test could quantify
   i. hepatic iron overload or liver fat (via PDFF maps) or iron (via T2* maps)
   ii. Levels of sensitivity and specificity in the parameters

g. Do you think the data from the MRCP+ might be impactful in treatment decision-making if used in standard care?

[Probe if the use of MRCP+ would alter treatment decisions if used in standard care?]

6) Individual perspective

Please could you provide some insights on your potential willingness to adopt MRCP+ by providing views and scores on the following statements about the MRCP+ service in management of PSC patients

Assessment of perceptions of using quantitative MRCP	Score	Rationale
Quantitative MRCP is a noninvasive diagnostic technique		
Quantitative MRCP facilitates the reporting of whole biliary tree metrics		

Quantitative MRCP suppresses noise and provides quantitative, visually rich models of the biliary tree from routine 3D MRCP		
Quantitative MRCP reports report whole tree metrics, such as duct number, biliary tree volume and gallbladder volume		
Quantitative MRCP reports report single duct metrics such a stricture and dilation length and number		
Quantitative MRCP allows the production of 2D graph of unfolded ducts, enabling easier monitoring of stricture(s)		
Quantitative MRCP allows for comprehensive assessment of the pancreaticobiliary tract morphology		
Quantitative MRCP allows for 3D visualisation which can aide improved characterisation of gallstones		
Quantitative MRCP is an accurate means for the early detection of PSC		
Quantitative MRCP is a way to accurately determine the stage of PSC which can support a prognosis		
Quantitative MRCP allows objective assessment of the biliary tract for monitoring of PSC progression		
Quantitative MRCP allows improved diagnosis and monitoring of PSC		
Quantitative MRCP is a clinically meaningful way to quantitatively define dominant bile stenosis		
Quantitative MRCP allows for enhanced visualisation of the biliary tree to support pre-surgery planning		
Quantitative MRCP is an effective tool for assessment of the biliary system pre- and post-liver transplant		
Quantitative MRCP is an accurate way to detect the early stages of cholangiocarcinoma		

7) Resourcing considerations

a. What would be the optimal mechanism to allow routine adoption of MRCP+ for monitoring of PSC patients?

[Probe which forms of funding might be used - Block contract, Tariff or alternative funding options- explain if possible?

Also would use in diagnosis be different

b. Do you consider that adopting MRCP+ testing in the care pathway could be facilitated within existing testing (resources - tariff)?

[Probe the tariff that might apply]

c. Does the involvement of an external organisation in the generation of the formulating the report have bearing on the option of a tariff funding?

d. Please could you comment on the potential price point and the implications for adoption

Note that the price points suggested for the MRCP+ analysis will be additional to the basic MRI cost of £116 to £133.00 per scan[1]

Option	MRCP+ charge per scan	Within tariff or requires uplift	Value Low / Acceptable / High
1	£100.00/scan		
2	£200.00/scan		
3	£300.00/scan		
4	£400.00/scan		
5	£500.00/scan		
7	£700.00/scan		
8	£1000.00/scan		

Reference: [1] NHS national tariff (2018/19)
