# Supplementary material for: Quantitative MRCP as Part of Primary Sclerosing Cholangitis Standard of Care in the National Health Service in England: A Feasibility Assessment Among Hepatologists
Source: Healthcare (Basel). 2025 Oct 20;13(20):2630. doi: 10.3390/healthcare13202630 (PMC12562387; doi:10.3390/healthcare13202630)
Supplement: Supplementary file 1 [file healthcare-13-02630-s001.zip › supplementary files/Supplementary Table S1.pdf]

Supplementary Table S1: Consensus points concerning standard of care raised by clinicians.

Diagnosis	• PSC staging is typically performed using MRCP, with ERCP used in cases of uncertainty/inconclusive results or low quality MRCP.<br>• The use of liver biopsy is at diagnosis is heterogenous between clinicians.
Monitoring	• Performed using a combination of blood and ultrasound tests (every 6-12 months), as well as MRCP (every 1-3 years). The frequency of testing can be increased depending on disease severity.<br>• ERCP is used only for intervention, there is a clinical indication, and is not part of routine monitoring.
Guidelines	• Clinician participants typically refer and aligned their practice to a combination of British (BSG, UK-PSC) and European (EASL) clinical practice guidelines.
Existing treatment	ERCP:<br>• Used therapeutically to dilate strictures in the biliary tree.<br>Pharmacotherapy:<br>• Ursodeoxycholic acid (UDCA) typically prescribed to patients with PCS, however, there is no evidence that it alters the natural history of PSC in adults.<br>• Corticosteroids and immunosuppressants are not used as treatments for PSC in adults but may be used in children and young adults who have overlapping features of autoimmune hepatitis (7-33%), or in those with concomitant inflammatory bowel disease (IBD) (>75%).<br>Liver transplants:<br>• The only lifesaving intervention for people with PSC, but disease can recur (de novo) in ~30%.<br>• Reserved for patients with persistent jaundice, decompensated cirrhosis (chronic liver failure) or recurrent acute cholangitis
Unmet need	• Surrogate endpoint: There are no (regulatory approved) surrogate endpoints to validate the efficacy of new and experimental therapy.<br>• Pharmacotherapy: No medical therapy is proven to slow disease progression in PSC.<br>• Paediatric management: Children and young adults diagnosed with autoimmune hepatitis first, with comorbid disease may have late diagnosis of PSC as comorbid disease is under-recognised outside specialist liver units.<br>• Cancer surveillance: Screening and surveillance for cholangiocarcinoma is suboptimal and there is a lack of evidence-based cancer surveillance strategies for PSC even though bile duct cancer develops in approximately 15% of patients.<br>• De novo PSC: Recurrence of PSC post-transplant (~30%) contributes to increased rates of graft loss and requiring re-transplantation.
Future trends for the next 3-5 years	Areas where quantitative MRCP can support:<br>• PSC management is an area with strong developmental potential as several phase II and phase III clinical trials are nearing completion, therefore there would likely be new therapies available in the coming 3-5years.<br>• Biliary complications account for a high percentage of adverse events following liver transplantation, prognostic markers that can predict these events will be highlight sought after to support patient management.
