# Supplementary material for: Quantitative MRCP as Part of Primary Sclerosing Cholangitis Standard of Care in the National Health Service in England: A Feasibility Assessment Among Hepatologists
Source: Healthcare (Basel). 2025 Oct 20;13(20):2630. doi: 10.3390/healthcare13202630 (PMC12562387; doi:10.3390/healthcare13202630)
Supplement: Supplementary file 1 [file healthcare-13-02630-s001.zip › supplementary files/Supplementary Table S2.pdf]

Supplementary Table S2: Consensus points concerning the use of quantitative MRCP+ as part of standard of care raised by clinicians.

Diagnosis	• The quantitative report allows quantitative and objective measures as opposed to the current subjective measure of MRCP.<br>• The quantitative report can provide a better understanding of the biliary tree which can help to diagnose and exclude PSC in patients more accurately.<br>• Data showing the incremental benefit of using quantitative MRCP+ alongside traditional MRCP in diagnosis are required.
Monitoring	• Monitoring using MRCP is subjective, a more quantitative and reproducible approach using MRCP+ will be more helpful as objective evidence will be provided over a longitudinal period.<br>• Objective assessment of the biliary tree and associated ducts using MRCP+ metrics will provide a more precise level of progression and staging which can support identification of adequate therapeutic response.<br>• Longitudinal objective measurement of stricture and dilatations of concern over time can support better understanding of disease progression and support better staging.<br>• Quantitative MRCP can support treatment planning, from the point of view of the endoscopist in the cases with fast stricture progression.<br>• It Is likely to be used more frequently in patients with high (annually) and intermediate (1-3 years) risk, compared to those with low-risk (3-5 years) risk.
Guide to liver transplants	• MRCP+ has shown evidence to predict PSC outcomes, therefore:<br>o It may have the potential to early identification of patients in need of transplant; these studies should be conducted.<br>o There is strong potential to monitor biliary tract post-transplant and to determine whether someone needs a resection of the bile ducts.<br>• There is the potential to show the degree of bile duct damage, which can help prioritise and stratify at-risk patients most likely in need a transplant in later life.<br>• MRCP+ would not be used to guide transplantation but will ideally be useful as an additional tool alongside clinical examination, evidence of portal hypertension, evidence of recurrent cholangitis or persistent jaundice, and abnormal blood tests.<br>• Similar to showing superiority over PSC scores (ANALI and MAYO risk score and Amsterdam-Oxford model), for use in transplantation MRCP+ will need to show superiority to the current UK Model for United Kingdom Model for End-Stage Liver Disease (UKELD) system used to score patients for a transplant.
Other indications	Looking beyond autoimmune liver disease, quantitative MRCP may have utility in:<br>• Pancreatic disease including assessment of the pancreatic duct in patients with chronic pancreatitis or lesion assessment in patients with IPMNs.<br>• Detection of gallstones within the bile ducts in patients presenting with acute abdominal pain and deranged liver function tests.<br>• Monitoring patients for biliary complications following liver transplantation
